# Identification of Differentiating Metabolic Pathways between Infant Gut Microbiome Populations Reveals Depletion of Function-Level Adaptation to Human Milk in the Finnish Population

**DOI:** 10.1128/mSphereDirect.00152-19

**Published:** 2019-03-20

**Authors:** Jan Majta, Krzysztof Odrzywolek, Bozena Milanovic, Vladyslav Hubar, Sonia Wrobel, Emilia Strycharz-Angrecka, Szymon Wojciechowski, Kaja Milanowska

**Affiliations:** a Ardigen, Krakow, Poland; b Department of Computational Biophysics and Bioinformatics, Faculty of Biochemistry, Biophysics and Biotechnology, Jagiellonian University, Krakow, Poland; c Department of Computer Science, Faculty of Computer Science, Electronics and Telecommunications, AGH University of Science and Technology, Krakow, Poland; d Department of Biophysics, Faculty of Biochemistry, Biophysics and Biotechnology, Jagiellonian University, Krakow, Poland; e Faculty of Biochemistry, Biophysics and Biotechnology, Jagiellonian University, Krakow, Poland; f Department of Medical Physics, Institute of Physics, Astronomy and Applied Computer Sciences, Jagiellonian University, Krakow, Poland; University of Wisconsin—Madison; Wroclaw University of Environmental and Life Sciences; Bromberg Lab, Rutgers University

**Keywords:** bioinformatics, gut microbiome, infant microbiome, machine learning, microbiome

## Abstract

A variety of autoimmune and allergy events are becoming increasingly common, especially in Western countries. Some pieces of research link such conditions with the composition of microbiota during infancy. In this period, the predominant form of nutrition for gut microbiota is oligosaccharides from human milk (HMO). A number of gut-colonizing strains, such as *Bifidobacterium* and *Bacteroides*, are able to utilize HMO, but only some *Bifidobacterium* strains have evolved to digest the specific composition of human oligosaccharides. Differences in the proportions of the two genera that are able to utilize HMO have already been associated with the frequency of allergies and autoimmune diseases in the Finnish and the Russian populations. Our results show that differences in terms of the taxonomic annotation do not explain the reason for the differences in the *Bifidobacterium*/*Bacteroides* ratio between the Finnish and the Russian populations. In this paper, we present the results of function-level analysis. Unlike the typical workflow for gene abundance analysis, BiomeScout technology explains the differences in the *Bifidobacterium*/*Bacteroides* ratio. Our research shows the differences in the abundances of the two enzymes that are crucial for the utilization of short type 1 oligosaccharides.

**IMPORTANCE** Knowing the limitations of taxonomy-based research, there is an emerging need for the development of higher-resolution techniques. The significance of this research is demonstrated by the novel method used for the analysis of function-level metagenomes. BiomeScout—the presented technology—utilizes proprietary algorithms for the detection of differences between functionalities present in metagenomic samples.

## Abstract (precis)

Knowing the limitations of taxonomy-based research, there is an emerging need for the development of higher-resolution techniques. The significance of this research is demonstrated by the novel method used for the analysis of function-level metagenomes. BiomeScout—the presented technology—utilizes proprietary algorithms for the detection of differences between functionalities present in metagenomic samples.

## INTRODUCTION

Depletion of oligosaccharides from human milk (HMO)-adapted bifidobacterial strains reduces their ability to dominate the gut microbiota in the presence of human milk. It allows other taxa, e.g., *Bacteroides*, to reach the dominant position. The lack of some strains of the *Bifidobacterium* genus has been linked by other researchers with the presence of more genes that encode virulence factors. Additionally, the extensive growth of *Bacteroides* has been described as a potential factor for the development of allergies and autoimmunity. Therefore, the described findings support the hypothesis that the depletion of HMO-utilizing bifidobacterial strains causes low levels of *Bifidobacterium* in Finnish infants’ microbiota.

### Importance of the gut microbiome.

Understanding of the role of commensal microbiota in human health and well-being has developed over the past few years (1–3). The gastrointestinal (GI) tract community is the most deeply investigated microbiome of the human body. A number of pieces of research have indicated its role in the development of various diseases, including but not limited to GI-related conditions like irritable bowel disease (4, 5) or Crohn's disease (6–8). Recent studies have shown the impact of commensal microbiota on the immune system in processes like cancer immunotherapy (9–11) or the development of autoimmune conditions (12–14). Nowadays, a variety of autoimmune and allergy events are becoming increasingly common (15). A well-established hygiene hypothesis connects such diseases with life conditions during the developmental period of life (16). One proposed hypothesis that explains the molecular background of autoimmunity and allergy development is that GI bacteria stimulate the immune system during early childhood (14).

It has now been demonstrated that the compositions of GI microbiota differ in the various populations of infants in the north European cohort. A study by Vatanen et al. showed that *Bacteroides* are a major genus in the Finnish population, while *Bifidobacterium* spp. dominate Russian guts. Nonetheless, it remains unsolved why such a difference occurs as both clades are present in both groups but with different abundances. A potential explanation for such a phenomenon may be the fact that the *Bifidobacterium* strains that colonize the Finnish population do not act as strong HMO metabolizers. Due to poor adaptation to small type 1 oligosaccharides, the GI tracts of infants may be not an optimal niche for them (17). On the other hand, some *Bacteroides* strains are able to take advantage of the presence of some HMOs and outgrow competing *Bifidobacterium* (18).

### Infant gut microbiota characteristics.

Various studies have reported a spectrum of variants of the composition of GI microbiota that are typical of infants (19). Despite these differences, the bacterial genera that are present in most research are *Bifidobacterium*, *Bacteroides*, *Escherichia*, and *Lactobacillus* (20). Multiple pieces of research have reported an association between the relative abundances of clades and various development conditions.

The infant microbiome profile relies on multiple factors. The most important are delivery type, antibiotic usage in both mother and infant, and formula feeding (21–23). The initial colonization of the infant takes place during and shortly after delivery as different communities are transferred from the mother’s body depending on whether birth is performed via vaginal delivery (predominantly vaginal lactic acid bacteria) or C-section (predominantly skin-bound *Staphylococcus*) (24). Furthermore, the history of the mother’s antibiotic usage determines the communities that may be transferred to a newborn (23).

During the 20th century, the composition of the GI microbiota of infants gradually changed. This can be noticed in comparisons of the pH levels of infant stool samples reported in a number of publications from the 20th century. Additionally, analyzing historical descriptions of the fecal microbiome, it can be easily noticed that there are clear differences (25) between the preantibiotic era and current research.

It seems that of the multiple species that are commensal to infants, *Bifidobacterium* spp. have the strongest association with infants’ health. Additionally, a significant amount of research reports that *Bifidobacterium* spp. represent the original inhabitants of infants’ GI tract (25–27). It was already proven that the presence of specific *Bifidobacterium* bacterial strains is correlated with a low level of virulence factor genes in the whole microbiota of infants (28).

The research by Vatanen et al. on the DIABIMMUNE (http://www.diabimmune.org/) cohort showed differences in autoimmunity and allergy events. The paper concluded that differing compositions of GI microbiota are responsible for this difference (14).

### HMO as a key prebiotic.

The primary food of a newborn is breast milk. Apart from water, breast milk is mostly composed of carbohydrates, and the predominant sugar present in breast milk is lactose (around 70 g/liter) (29). Breast milk also contains indigestible oligosaccharides that act as prebiotics for coevolved GI microbiota members—human milk oligosaccharides (HMOs)—that are present with an approximate concentration of 20 g/liter. HMOs can be primarily divided into two groups: type 1 oligosaccharides, e.g., lacto-n-biose or lacto-n-tetraose, which are characterized by Galβ1-3GlcNAc linkage, and type 2 HMOs, which possess Galβ1-4GlcNAc linkage (17). What is specific only to human milk is that type 1 chain oligosaccharides are predominant in the mixture (17, 30).

While present in breast milk with a full spectrum of different molecules, HMOs were originally not a primary objective of formula manufacturing. Currently, different oligosaccharide mixtures are being added to formula to mimic the presence of HMO (31); however, most formulas on the market do not offer full mimicking of a rich HMO composition.

The ability of certain strains of *Bifidobacterium* to utilize some HMOs seems to be an evolutionary adaptation to GI tract conditions. Additionally, these strains tend to possess enzymes specific to type 1 HMOs (32). Studies on breast-fed and formula-fed infants show different compositions of the GI microbiota of infants, with a decrease of *Bifidobacterium* within formula-fed groups (33).

### *Bacteroides* and *Bifidobacterium* species utilize HMOs in different pathways.

A variety of bacterial species are reported to grow on a medium with HMOs. In addition to *Bifidobacterium*, *Bacteroides* species have also been reported to exhibit growth on a full HMO spectrum (34). Comparison between the species growth dynamics of *Bifidobacterium* and *Bacteroides* shows that *Bifidobacterium* spp. are able to grow faster than *Bacteroides*; thus, they become a dominant community member (18).

Analyses of growth on different HMO fractions have shown that short HMOs are utilized exclusively by some strains of *Bifidobacterium* (e.g., B. longum subsp. *infantis*, B. bifidum). On the other hand, more-complex HMOs are metabolized by a number of species, including gut-inhabiting Bacteroides fragilis (18).

The proposed source of this difference is the fact that *Bifidobacterium* spp., which coevolved with GI tract conditions, are able to adapt to the utilization of the HMOs that are present in the intestines of breast-fed infants. This hypothesis is supported by the fact that the major HMO molecules that are present in human milk are of short chain type 1. *Bacteroides* spp., on the other hand, utilize longer type 2 HMOs by using the mucin glycan-degrading pathway. Due to the similarity of host mucin glycans to some HMOs, *Bacteroides* are able to utilize a large fraction of breast milk; however, the ability of *Bacteroides* to grow with HMOs as a carbon source has limitations due to the fact that the HMO proportion in human milk is better suited to *Bifidobacterium* (18, 35).

Utilization of HMOs depends on a number of enzymes. The most general HMO-related enzymes are collocated in the so-called HMO cluster that is present in a number of *Bifidobacterium* species. The members of this cluster that are used for utilization of HMOs are beta-galactosidase, alpha-fucosidase, and sialidase, transporters that allow the transport of predigested oligosaccharides to bacterial cells and other proteins (36).

In addition to the enzymes that are common to a number of species, there are a variety of strain-specific enzymes that play key roles in certain metabolic pathways. B. longum subsp. *infantis*—the most-studied HMO-metabolizing strain—possesses additional β-galactosidase—lacto-n-biosidase (LNB)-β-galactosidase (Bga42A)—that is not collocated with other HMO cluster genes. This enzyme is used exclusively to utilize short type 1 HMOs as it has high specificity for lacto-N-tetraose (LNT) (37).

The same phenomenon is observed in B. bifidum—another HMO-utilizing bacterial species that possesses a metabolic pathway for type 1 HMOs and uses specific LNB (38).

As both *Bifidobacterium* and *Bacteroides* spp. are able to utilize breast milk prebiotics, it seems that different strains of each genus, each with different modes of metabolism, are present in both populations. The research by Vatanen et al. described equal abundances of HMO cluster genes in both groups. The aim of our study was to detect the genomic features associated with the strain-specific enzymes crucial for short type 1 HMO metabolic pathways and to explain the differential abundances of both HMO-utilizing genera in the Finnish and the Russian group.

## RESULTS AND DISCUSSION

### Taxonomy-based analyses.

**FIG 1:**
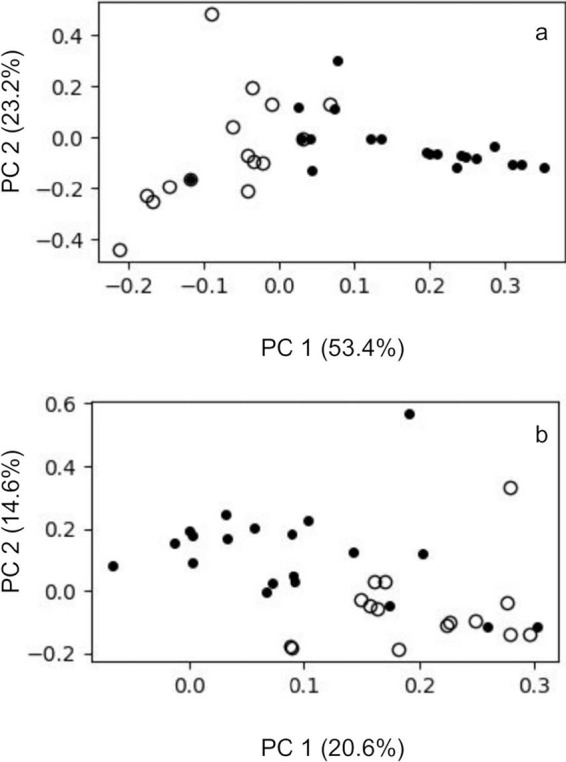
Principal-coordinate analysis done for the taxonomy profiling results of all the samples, with the Russian (solid dots) and Finnish (open circles) groups done with genus-level taxonomy assignment and deepest-achieved-taxonomy assignment.

As reported by Vatanen et al., the Russian and Finnish groups can be characterized by their different compositions of GI microbiota. Data from principal-coordinate analysis (PCA) done on the genus level (Fig. 1a) and on the “deepest achieved” taxonomic level (Materials and Methods and Fig. 1b) show separation of the Finnish and Russian groups; nevertheless, no significant difference in GI microbiota diversity was detected. Operational taxonomic unit (OUT) richness analyzed for both the deepest achieved taxonomic level and the genus level was higher in the Finnish group (*P* = 0.02 and *P* = 0.01, respectively). However, exclusion of extremely rare (<0.005% abundance) taxa resulted in insignificant differences in OTU richness (*P* = 0.68 and *P* = 0.25 for the deepest achieved taxonomic level and genus level, respectively). While richness is highly dependent on sequencing depth and the actual composition of the community, inclusion of Shannon index allows better understanding of a sample’s diversity. The Shannon index (39) values do not differ significantly between populations, both at the genus level (*P* = 0.8) and at the deepest achieved level (*P* = 0.77).

**FIG 2:**
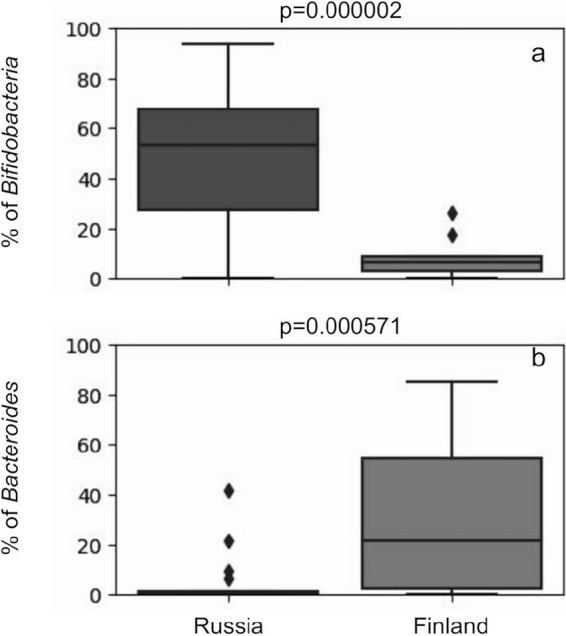
Relative abundances of *Bifidobacterium* (a) and *Bacteroides* (b) genera in samples of the Russian (left) and the Finnish (right) groups.

The major differences between the groups were the levels of *Bacteroides* and *Bifidobacterium* (*P* < 0.005) (Fig. 2; see also Table S1 in the supplemental material). Finnish infants were mostly colonized by *Bacteroides* species, while the Russian infants were hosts for a variety of *Bifidobacterium* species.

10.1128/mSphereDirect.00152-19.1 TABLE S1 Abundance table for each analyzed sample, with raw results and results collapsed at the genus level. Download Table S1, XLSX file, 1.4 MB. Copyright © 2019 Majta et al. 2019 Majta et al. This content is distributed under the terms of the Creative Commons Attribution 4.0 International license.

However, while *Bacteroides* species are rare in the Russian population (1.3% abundance at the 75th percentile), the GI microbiome composition in the Finnish population contains a significant amount of *Bifidobacterium* spp. (8.7% abundance at the 75th percentile). It can be clearly seen that despite their GI tracts being dominated by *Bacteroides*, a significant amount of *Bifidobacterium* spp. is present in the Finnish population. Therefore, it is not the lack of colonization of infants’ GI tract by members of *Bifidobacterium* genus that results in the dominance of *Bacteroides*. Moreover, the levels of major clades (*Bifidobacterium* and *Bacteroides*) differ in the Russian and Finnish populations, respectively. While *Bifidobacterium* reaches 69.7% at the 75th percentile in the Russian population, the Finnish infants have 58% *Bacteroides* at the 75th percentile. Additionally, as reported by Vatanen et al., differences in *Bifidobacterium* abundance within a cohort should not be related to differences in breastfeeding duration, as the breastfeeding period in the Russian group was shorter. The reported abundance analyses show that *Bifidobacterium* gut-colonizing strains are stronger colonizers of infants’ GI tracts than *Bacteroides* strains. Moreover, the presence of a significant amount of *Bifidobacterium* in the Finnish population may support the hypothesis that the strains that colonize these infants are different from those colonizing Russians. However, observations of such taxonomic differences require function-level evidence to fully support this hypothesis.

### Genomic feature-based analysis.

**FIG 3:**
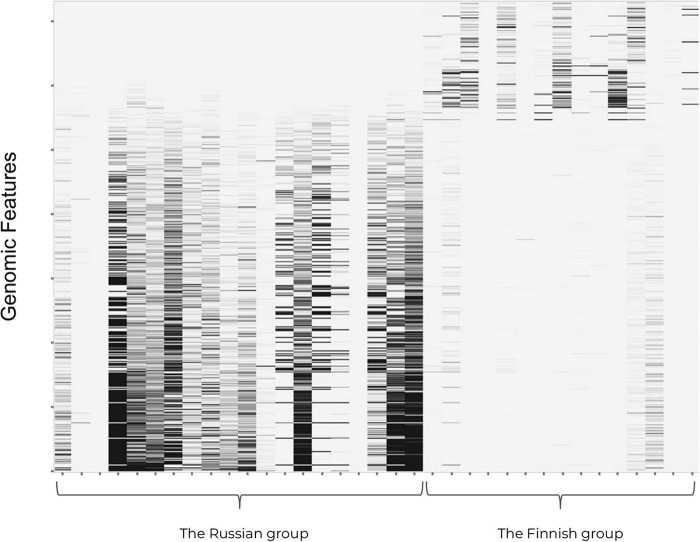
Heat map showing a normalized BiomeScout score level in samples for selected (*P* < 0.05) genomic features. Genomic features were sorted by an average normalized BiomeScout score in the Russian group (highest at the bottom).

Genomic features were extracted from a data set comprising approximately 400 M reads in 35 samples (see Materials and Methods). The constructed feature space had roughly 1.2 M genomic features. The BiomeScout Feature Selection module selected ∼50,000 genomic features, all of which were significantly (*P* < 0.05) enriched in one but not both of the groups. In contrast to the lack of differences in compositional diversity between groups (shown by comparison of Shannon indices of samples from the Russian and the Finnish groups), the majority of enriched genomic features were found in the Russian population (Fig. 3).

Detection of the majority of selected genomic features that were enriched in the Russian population suggests the presence of unique genomic components in genomes of members of that population’s commensals. We also noticed that there are very few genomic features that are specific to microbes that inhabit the guts of Finnish infants. The fact that there were relatively few unique genomic features in the Finnish guts made it possible to infer that the majority of bacteria present there were highly similar to those that colonize the GI tract of Russians.

Comparison of these findings with data from taxonomy-based compositional analysis suggests that the Russian microbiome-specific genomic features may come from specific strains of *Bifidobacterium* that overgrow other clades. This is in accordance with the previous description of the breast milk-boosted growth of *Bifidobacterium* (17).

### Functional analysis.

For the functional analysis, all genomic features were associated with the known bacterial genes. Annotation was performed with regard to the sequence comparison with a curated UniProtKB database (http://uniprot.org/).

The analysis of selected genomic features showed that a number of them were associated with HMO-processing functionalities (Table 1). Genes known to be collocated within a bifidobacterial HMO cluster (fucosidase, sialidase, galactosidase) were associated with the multiple genomic features enriched in the Russian population.

**TABLE 1:** List of top scored genomic features significantly differentiating in two populations (the Russian and the Finnish) that were associated with proteins playing key role in HMO metabolism (located in bifidobacterial HMO cluster)

Genomic feature ID^*a*^	*P* value	Annotated name
SRX2234520_64394	0.005	6-Phospho-beta-galactosidase
SRX2199553_754	0.005	Beta-galactosidase; evolved beta-galactosidase subunit alpha
SRX2199674_8463	0.007	Beta-galactosidase; evolved beta-galactosidase subunit alpha
SRX2199674_13733	0.007	6-Phospho-beta-galactosidase
SRX2199763_1108	0.022	Alpha-l-fucosidase 2; probable alpha-fucosidase A
SRX2199670_2849	0.024	Alpha-l-fucosidase 2; probable alpha-fucosidase A
SRX2199670_12138	0.024	Probable alpha-fucosidase A
SRX2199300_19278	0.024	Probable alpha-fucosidase A
SRX2199674_15004	0.012	Sialidase; exo-alpha-sialidase
SRX2199674_12906	0.015	Exo-alpha-sialidase; sialidase-1
SRX2199670_6933	0.016	Sialidase
SRX2199553_8800	0.025	Sialidase
SRX2199608_26754	0.033	*N*-Acetylneuraminate lyase
SRX2234520_48491	0.041	*N*-Acetylneuraminate lyase
SRX2234453_7550	0.043	*N*-Acetylneuraminate lyase

a ID, identifier.

**FIG 4:**
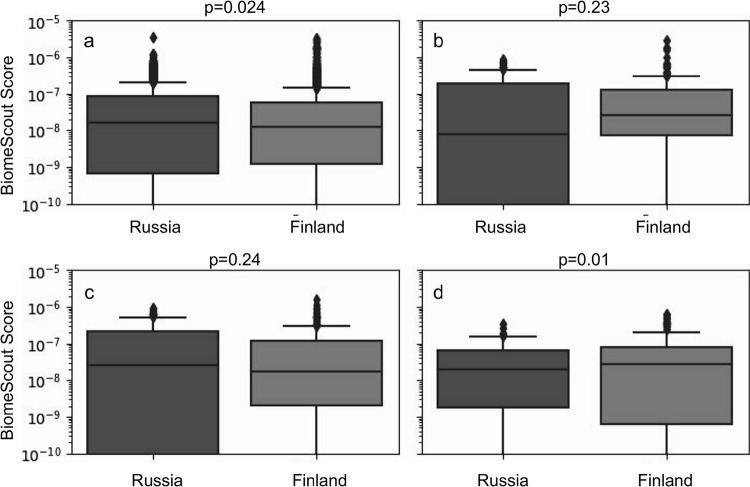
BiomeScout scores for enzymes present in the HMO cluster. Mean values for those enzymes were approximately equal for the Russian and the Finnish groups and were as follows: for beta-galactosidase, 8.1e−8:8.7e−8 (a); for alpha-fucosidase 1.3e−7:1.8e−7 (b); for sialidase 1.4e−7:1.1e−7 (c); for *N*-acetylneuraminate lyase, 4.5e−8:7.5e−8 (d). All genomic features detected in the whole cohort were used.

Apart from the fact that such functionalities were associated with the genomic features typical of Russian infants, the same functionalities were also associated with a number of other genomic features. Consequently, these functional annotations revealed the presence of approximately equal levels of genomic features that are related to genes corresponding to the HMO cluster in the Russian and Finnish populations (Fig. 4).

Detection of genomic features associated with the genes present within the HMO cluster and of equal distributions of them in the two populations is consistent with the analysis by Vatanen et al. In their 2016 paper (14) on the same cohort, they reported such equality with regard to HMO cluster genes. In the same research, they found that such genes are mostly present in *Bacteroides* within the Finnish population and in *Bifidobacterium* within the Russian population. Such findings are consistent with our findings based on the BiomeScout technology, but taxonomy-agnostic genomic features do not imply the source organism at this step of analysis.

### Detection of enzyme enrichment.

To verify the hypothesis that the bifidobacterial strains present in the Russian population possess functionalities typical of enzymes reported as strain specific, we analyzed different HMO-processing enzymes. We chose two enzymes that are key for the utilization of the type 1 HMOs that are most common in human milk in the two key bifidobacterial species: lacto-n-biosidase (LNB) of B. bifidum and β-galactosidase from B. longum subsp. *infantis* (Bga42A).

As was already noticed in the case of HMO cluster genes, analyses of significantly enriched genomic features may be misleading; therefore, all genomic features were used in the analysis of strain-specific functionalities. Among these features, we chose those associated with specific HMO-processing enzymes: LNB and Bga42A (Materials and Methods).

**FIG 5:**
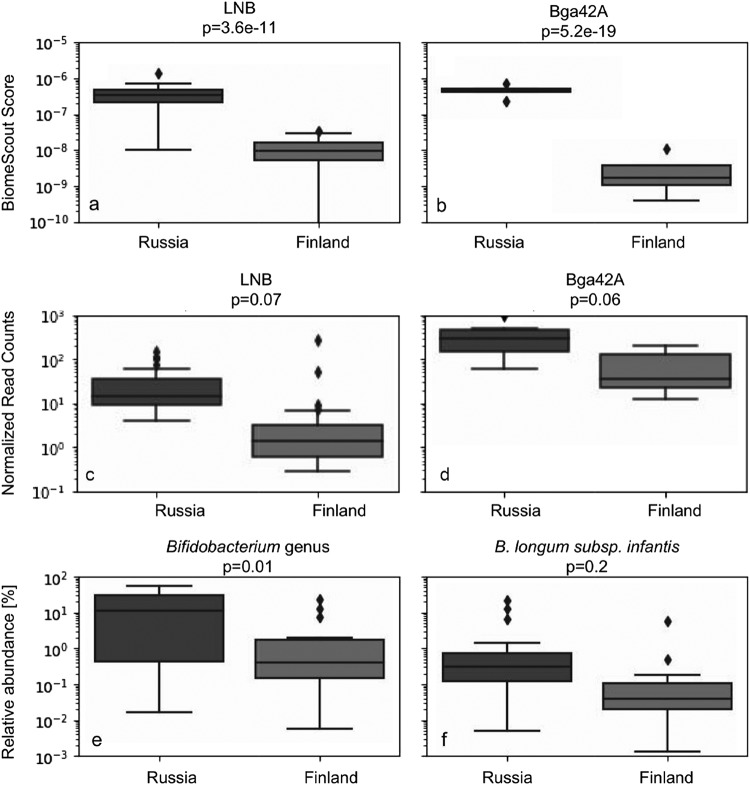
BiomeScout scores of all genomic features annotated as matching lacto-N-biosidase (a) and beta-galactosidase Bga42A (b) significantly differentiate the Russian and the Finnish groups. In contrast, the counted reads mapped against those genomic features (c and d, respectively) were shifted in the same way, but the difference was insignificant. Differential presence of Bga42A in samples cannot be inferred from the abundance of B. longum subsp. infantis. The strain that contains this enzyme was not detected with significantly higher abundance in the Russian population (f), while bacteria of the *Bifidobacterium* genus were significantly more abundant (e).

BiomeScout analysis showed a significantly higher level of genomic features associated with gene-encoding enzymes specific to short type 1 HMOs in the Russian population. Genomic features associated with lacto-n-biosidase, present predominantly in B. bifidum and some bifidobacterial strains, were found to be significantly enriched (*P* = 3.6e−11) in the Russian population (Fig. 5a).

Additionally, genomic features associated with Bgal42A that are present specifically in B. longum subsp. *infantis* were found to be present at a significantly higher level (*P* = 5.2e−19) in that population (Fig. 5b).

Differences in the genomic features associated with short type 1 HMOs reveal the basis of the differences between the *Bifidobacterium* abundances in the guts of Russian and Finnish infants. *Bifidobacterium* strains that lack the ability to utilize short type 1 HMOs have to compete with other community members that are able to utilize a broad HMO fraction, mostly *Bacteroides*. Additionally, over 26% of the whole human HMO composition consists of LNT (40); thus, the lack of ability to metabolize this compound is a significant handicap. As described by Marcobal et al. (18), under conditions of use of porcine mucin glycans as a sole source of carbon, *Bacteroides* overgrow *Bifidobacterium*. The proposed explanation for such a difference was the fact that the compounds in a large fraction of HMO are similar to mucin glycans. *Bacteroides* spp. use such oligosaccharides as their main carbon source, while *Bifidobacterium* spp. are adapted to the use of the small oligosaccharides of human milk. Therefore, our findings are in accordance with the growth dynamics on short type 1 HMOs, complex glycans, and full HMOs that were described by Marcobal et al.

Such a finding shows the possibility of detecting enzyme-specific function-level differences between two populations. Such an analysis, performed with a straightforward pipeline that counts reads mapped to each reference gene, shows no significant differences between the two groups in the levels of these genes (Fig. 5c and d).

Enrichment of LNB could be inferred from analysis of the taxonomic content of samples, as the Russian group was significantly enriched with B. bifidum (averages of 15.8% and 0.8% in the Russian and Finnish groups, respectively, *P* = 0.003), which possesses such an enzyme. Importantly, in the case of Bga42A from B. longum subsp. *infantis*, genomic feature-based analysis showed differences that were not revealed by the traditional taxonomy-based approach. Distinguishing B. longum subspecies—even with shotgun sequencing data—is an extremely complex task because these bacteria may share over 99% of genomic sequences. The fractions of microbiome members assigned as B. longum species and of those specifically assigned as B. longum subsp. *longum* were significantly enriched in the Russian population (*P* = 0.01 and *P* = 0.03, respectively). However, no significant difference in B. longum subsp. *infantis* abundance was detected between the Russian and the Finnish populations (*P* = 0.2) (Fig. 5e and f). Importantly, strain level was annotated only rarely.

In this paper, we report the ability to determine enzyme-scale differences between two populations using next-generation sequencing data. What is more, our approach makes it possible to detect differences that are hard to detect with standard pipelines for both taxonomy analysis and gene catalog annotation.

Importantly, BiomeScout analysis is done with no prior taxonomy assignment analysis –Genomic feature data are extracted from the raw sequencing data. Therefore, we were able to also utilize reads of ambiguous origin without bias toward information stored in the available databases.

The described use case allows detection of the differences between the bifidobacterial strains that colonize the Russian and the Finnish infants’ GI tract in terms of the presence of enzymes specific to short type 1 HMOs. As such oligosaccharides represent a large portion of the whole HMO mixture, we hypothesize that the lack of dedicated enzymes in the strains colonizing the Finnish infants allows *Bacteroides* to overgrow *Bifidobacterium*. Further studies are necessary to more deeply investigate the phenotypic differences between the dominating and dominated *Bifidobacterium* strains.

## MATERIALS AND METHODS

The data set analyzed in this study represents a subset of the DIABIMMUNE cohort (http://www.diabimmune.org) (14). The chosen samples were sequenced during the first 180 days of the infants’ lives. Infants from the DIABIMMUNE cohort were subsampled such that the groups were balanced. Sample collection in the Russian population started later (around month 3 of life); therefore, the Finnish samples from the earlier period of life were excluded from analyses. For each cohort member, only the sample from the first time point was included in the analysis. Only the Finnish and the Russian groups were chosen, as the Estonian population has previously been described as being in a “transition zone” between west and east due to political changes over the last 30 years. For a better understanding of early colonizing strains, only samples collected within the first 180 days of life were used for this study. During this period breast milk is the primary or only nutrition for infants. Additionally, such a period is crucial for the development of the immune system of infants. Consequently, 35 shotgun metagenomic sequencing samples were chosen for further investigation.

Taxonomy binning and profiling were done with Kraken2 (https://ccb.jhu.edu/software/kraken2/) (41). For binning of reads and taxonomy profiling of samples, a standard database was built according to the tool’s manual. Lineages were built for all assigned taxonomies with seven canonical levels (kingdom, phylum, class, order, family, genus, and species) and subspecies if assigned. Such annotation is referred to as the deepest achieved taxonomic level. Given the fact that multiple lineages were shorter than the subspecies level, lineages truncated above the genus level were also used. The abundances corresponding to the resulting identical assignments were summed.

Initial assembly of metagenomic samples was done with Megahit v1.1.3 (https://github.com/voutcn/megahit/) (42). Genomic feature-based analysis was done with BiomeScout tool v.1.0.2. In BiomeScout’s nomenclature, “genomic feature” is a general term that refers to any part of a genomic sequence. To construct the genomic feature space, BiomeScout used preassembled metagenomes from the whole studied cohort as an input-only data set.

Each feature was scored with respect to the two methodologies: counting mapped reads and BiomeScout. In the first, all reads mapped on each genomic feature were counted. BiomeScout analysis used its own internal proprietary scoring algorithm. The two scoring results were normalized with respect to the number of sequenced reads.

The scored genomic features were then passed to the feature selection algorithms, which choose a set of genomic features that significantly differentiate groups. Each selected genomic feature had a *P* value assigned by BiomeScout’s Feature Selection module using Student's *t* test. The same test was used in the whole analysis where *P* values are reported.

Functional annotation was done with bioinformatics analysis of each genomic feature. The similarity of the sequence to full or partial sequences of known proteins was calculated. Reviewed entries from the UniProtKB v. 2018_09 database (http://uniprot.org) (43) were used. Each genomic feature was matched with a set of known genes using DIAMOND v0.9.22 (https://github.com/bbuchfink/diamond) (44). Identity threshold analysis was based on a paper by Addou et al., which describes 80% identity as a threshold for 100% consensus of a four-digit EC number (45, 46). The same percentage was chosen for query coverage threshold based on the UniRef database clustering approach (47, 48).
